# Editorial: Progress in diagnosis and treatment of hypothalamic & pituitary disorders

**DOI:** 10.3389/fendo.2025.1674324

**Published:** 2025-08-20

**Authors:** Krzysztof C. Lewandowski, Elżbieta Skowrońska-Jóźwiak

**Affiliations:** ^1^ Department of Endocrinology and Diabetes, Collegium Medicum Mazovian University of Plock, Plock, Poland; ^2^ Department of Internal Disease and Clinical Pharmacology, The Medical University of Lodz, Lodz, Poland

**Keywords:** pituitary, pituitary tumors, glucagon stimulation test, Synacthen test, Prader-Willi syndrome, cerebrospinal fluid

Diagnostics of pituitary function is demanding as accurate assessment must take into account pulsatile nature of hormone secretion, the presence of diurnal rhythms, phase of menstrual cycles as well the influence of other factors such as obesity or diabetes. Lack of standardized guidelines for application of pituitary function tests, makes it challenging in terms to ensure consistent and accurate testing and interpretation across different clinical settings. The glucagon stimulation test (GST) is widely used to assess growth hormone (GH) and cortisol secretion, nevertheless the precise mechanisms underpinning these hormonal responses remain unclear. In original research study Kawalec et al. [Determination of glucose cut-off points for optimal performance of glucagon stimulation test] emphasized the importance of glucose monitoring during GST in order to validate optimal condition for GH stimulation and support clinical decisions in GH deficiency management. They showed that glucose nadir below 3.33 mmol/l is the only biochemical biovariable linked with optimal GH secretion during GST (i.e. 100% sensitivity), whereas mechanisms responsible for cortisol secretion remain unclear. Having said that, it must be also understood that adequate stimulus for GH secretion may be obtained also in subjects who fail to reach the above-mentioned glucose nadir, while the lack of a clear mechanism responsible for cortisol secretion does not imply any inferiority of this tests for HPA axis. Furthermore, optimal cortisol cut-off points during GST are also discussed.

In study of Góralska et al. [Assessment of hypothalamic-pituitary-adrenal axis impairment and effects of hydrocortisone treatment in adults with Prader-Willi syndrome], performed retrospective analysis on adults with Prader-Willi syndrome and the authors found some impairment of hypothalamic-pituitary-adrenal (HPA) axis during 250 µg short Synacthen test (SST) in 47% of examined patients, where adrenal insufficiency was defined by failure to reach 500 nmol/l (18 µg/dl) cortisol cut-off at 30 minutes of SST. Such percentage might have been even higher, if tests more dedicated for pituitary function testing (such as GST, or Insulin Tolerance Test (ITT)) had been employed. The Authors suggested routine evaluation of HPA axis in such patients with Parder-Willi syndrome and emphasized the benefits of steroid replacement therapy in affected patients.

Pituitary tumors, while usually histologically benign, can present unique challenges due to their location and potential to either disrupt hormone production, or to produce hormone excess. Despite the ever growing number of published studies, there is still a need for diagnostic and predictive biomarkers of pituitary adenomas. Wang et al. in original study [Elucidating the causal links between plasma and cerebrospinal fluid metabolites and pituitary tumors: a Mendelian randomization analysis] identified in cerebrospinal fluid (CSF) as much as 27 metabolites that might be associated with the incidence of pituitary tumors, among which 3-dehydrocarnitine and acetylcarnitine are the most noteworthy. Though clinical significance of these findings remains to be established, the study demonstrates another opportunity to study the nature of pituitary tumors that might include CSF testing.

Lisiewicz et al. in their review [The prospective roles of exosomes in pituitary tumors] showed involvement of exosomes in the pathology of pituitary adenomas and their potential clinical applications. Exosomal micro RNA, circular RNA, long non-coding RNA and messenger RNA expression were altered in pituitary adenomas, correlating with tumor invasiveness, diagnosis, and prognosis.

Finally, Yao and Chen reviewed new perspectives of therapy of pituitary tumor with everolimus [Everolimus in pituitary tumor: a review of preclinical and clinical evidence], FDA-approved mTOR inhibitor, which not only suppresses the growth and proliferation of APT cells but also enhances their sensitivity to radiotherapy and chemotherapy. These observations are interesting given known difficulties in treatment of aggressive pituitary tumors particularly in cases of temozolomide resistance.

We hope that this Research Topic of Frontiers in Endocrinology entitled Progress in Diagnosis and Treatment of Hypothalamic & Pituitary Disorders would be stimulating for our Readers.

